# AGE-FRIENDLY MESSAGES AND IMAGES: A VIEW FROM HEALTH PROFESSIONS EDUCATION

**DOI:** 10.1093/geroni/igac059.1065

**Published:** 2022-12-20

**Authors:** Marilyn Gugliucci

**Affiliations:** University of New England College of Osteopathic Medicine, Biddeford, Maine, United States

## Abstract

The World Health Organization (WHO) report published March 2021 emphasizes that older adults are often subjected to a variety of negative stereotypes including helplessness, frailty, and child-like qualities. Ageism has both real mental and physical health consequences, including a decreased will to live, less desire to live a healthy lifestyle, an impaired recovery from illness, increased stress, and a shortened life span. Health professions education has a responsibility to prepare future providers in more than mindful physical care of older adults, it must address ageism that has proliferated negative personal biases that have triggered reduced overall health and quality of life. Preparation of lectures and learning materials in health professions education requires mindfulness of diversity as well as the implicit bias faculty may portray regarding age. This presentation will bring these nuances to light for consideration and as a reference to instill change.

